# Optimization of preventive health care facility locations

**DOI:** 10.1186/1476-072X-9-17

**Published:** 2010-03-18

**Authors:** Wei Gu, Xin Wang, S Elizabeth McGregor

**Affiliations:** 1 Department of Geomatics Engineering, University of Calgary, 2500 University Drive NW, Calgary, Alberta, Canada, T2N 1N4; 2 Population Health Research, Alberta Health Services Cancer Care, 1331 - 29th Street NW, Calgary, Alberta, Canada, T2N 4N2

## Abstract

**Background:**

Preventive health care programs can save lives and contribute to a better quality of life by diagnosing serious medical conditions early. The Preventive Health Care Facility Location (PHCFL) problem is to identify optimal locations for preventive health care facilities so as to maximize participation. When identifying locations for preventive health care facilities, we need to consider the characteristics of the preventive health care services. First, people should have more flexibility to select service locations. Second, each preventive health care facility needs to have a minimum number of clients in order to retain accreditation.

**Results:**

This paper presents a new methodology for solving the PHCFL problem. In order to capture the characteristics of preventive health care services, we define a new accessibility measurement that combines the two-step floating catchment area method, distance factor, and the Huff-based competitive model. We assume that the accessibility of preventive health care services is a major determinant for participation in the service. Based on the new accessibility measurement, the PHCFL problem is formalized as a bi-objective model based on efficiency and coverage. The bi-objective model is solved using the Interchange algorithm. In order to accelerate the solving process, we implement the Interchange algorithm by building two new data structures, which captures the spatial structure of the PHCFL problem. In addition, in order to measure the spatial barrier between clients and preventive health care facilities accurately and dynamically, this paper estimates travelling distance and travelling time by calling the Google Maps Application Programming Interface (API).

**Conclusions:**

Experiments based on a real application for the Alberta breast cancer screening program show that our work can increase the accessibility of breast cancer screening services in the province.

## Background

Preventive health care programs aim to save lives and contribute to a better quality of life by diagnosing serious medical conditions early and reducing the likelihood of life-threatening disease. Evidence shows that successful treatment of some health problems is more likely if an illness is diagnosed at an early stage. Thus, efficient and effective preventive health care services have been an integral part of many health care reform programs within the past two decades [1-3].

Facility location decisions are a critical element in strategic planning in preventive health care programs [4]. Previous research proves that facility location plays a key role in the success of preventive health care programs in terms of the participation rate. A survey by Zimmerman [5] finds that the convenience of access to a facility is a very important factor in a client's decision to have prostate cancer screening. Furthermore, a survey by Facione [6] reveals that perceptions of lack of access to services are related to a decrease in mammography participation. A recent review by Baron et al.[7] finds that the efficacy of reducing structural barriers (including distance required to travel to obtain mammograms) increases community access to breast, cervical, and colorectal cancer screening.

Characteristics of preventive health care services are inherently different from other health care services (such as health care for acute diseases), which requires a different location decision methodology. The first characteristic of preventive health services is that people might not seek services from the closest preventive health care facility. Since preventive services are given to people with no clear symptoms of illness, people who seek preventive services have more flexibility as to when and where to receive preventive health care services [2,3]. For example, for a person living in an area serviced by two preventive health care clinics within an acceptable travelling distance, the person may choose the closer one because of the convenience. Or he/she may go to the farther clinic, located near a shopping mall, because he/she can go shopping after a medical appointment. The second characteristic of preventive health services is that each facility needs to have a minimum number of clients to retain the accreditation, except when there is a policy decision to provide preventive services to sparsely populated neighborhoods. For example, the U.S Food and Drug Administration (FDA) requires a radiologist to interpret at least 960 mammograms and a radiology technician to perform at least 200 mammograms in 24 months to retain their FDA accreditation [8].

According to the report from the World Health Organization [9], current health care systems do not make optimal use of available resources to support preventive health care programs. One of the reasons is that the location of preventive health care facilities is determined without fully considering the above two characteristics. In the current health care systems most facilities are located based on responding to emergent medical problems, which assumes that people would seek services from the nearest facility. Thus, location optimization is performed based on the distance between people and their assigned closest facility [10].

In this paper, we present a methodology for the optimal location configuration of preventive health care facilities. In order to satisfy the characteristics of preventive health care services, we define the concept of accessibility to preventive health care services as the measurement for location optimization. The accessibility to preventive health care services used in this paper is comprised of three factors:

(1) Regional availability of preventive health care services. Regional availability is expressed as a ratio between clients and preventive health care facilities within a region. A client in a higher ratio region has more convenient access to services. Regional availability considers all of the facilities within an acceptable travelling distance of a client when calculating the accessibility of preventive health care services to that client. The assumption behind regional availability is that people may go to any facility within the acceptable travelling distance constraint, which satisfies the first characteristic of preventive health care services that people might not seek services from the closest preventive health care facility.

(2) Travelling distance between facilities and clients. The clients within an acceptable travelling distance of a facility do not share this facility equally since usage decreases with distance. The closer client would have higher accessibility to the facility. This factor satisfies the first law of geography [11], which states that "everything is related to everything else, but near things are more related than distant things" and the well-known fact that distance affects access to health care services [12].

(3) Each facility should attract a minimum number of clients unless the facility is located in a remote place. This factor satisfies the second characteristic of preventive health care services. We use the Huff-based competitive location model [13] to estimate the workload of facilities. The assumption behind the model is that the probability of a client getting service from a facility within the acceptable travelling distance constraint is related to two elements. The first element is the attraction of the facility. In this paper, the attraction of a facility is described by the inverse travelling distance between the facility and a client. The second element is the inverse of the sum of the attractions of all facilities within the acceptable travelling distance constraint, which means the more facilities that are located within an accessible distance of a client, the lower the chance that a particular facility will be used by the client.

In this paper, the accessibility of preventive health care services only focuses on structural barriers that are directly related to the number, concentration, and location of healthcare facilities. The financial barriers (e.g., availability of insurance coverage) and personal barriers (e.g., social and cultural aspects) [14] are not discussed. Additionally, in this paper, we only consider the configuration of preventive health care facilities. The potential interaction between preventive health care facilities with other facilities (i.e., primary health care facility) is not considered.

Based on the new definition of accessibility, this paper proposes a bi-objective model to optimize the location of preventive health care facilities. As appropriate for publically funded health care facilities, the optimizing objectives are to improve efficiency and coverage of the preventive health care facilities. The bi-objective model is solved using the Interchange algorithm [15]. To accelerate the solving process of the Interchange algorithm, two new data structures, 'population groups' and 'candidate string,' are implemented in order to pre-store the accessibility information.

Additionally, this paper uses travelling distance and travelling time to measure the spatial barrier between clients and preventive health care facilities. The travelling distance and travelling time are estimated accurately and dynamically by calling the Google Maps Application Programming Interface (API) [16]. The Google Maps API is a software program that defines how other software can request services (the same services we can get from the http://maps.google.com web page manually) from the Google. The Google Maps API is easier than the previous travelling time estimation methods [17,18] in that it does not need users to supply speed limit maps and gather traffic rules.

Finally, the methodology proposed in this paper is evaluated using a real application: optimizing the configuration of breast cancer screening services in Alberta, Canada. Experiments show that the methodology would help to increase the accessibility of breast cancer screening services in the province.

In the following sections we: 1) provide a sketch of relevant background literature; 2) formalize the problem in the paper with respect to the characteristics of preventive health care services and present a solution approach; 3) describe the procedure for applying the methodology to a real-world scenario, namely the Alberta breast cancer screening program; and demonstrate the effectiveness and efficiency of the methodology for this purpose; and finally 4) conclude the paper with a discussion of future research directions.

### Basic facility location models

The location of facilities is critical to the success of health care services [4]. Although the health care facility location problem has been studied for thirty years, the characteristics of preventive health care services have not been fully incorporated into the prevailing facility location models. In this subsection, three basic facility location models are introduced first, which are the foundations of most preventive health care facility location models. Three classic facility location models are: *the P-median model, the covering model *and *the center model *[19]. All three models assume that people would seek services from the closest facility. The models optimize facility locations based on the distance from clients to their closest facility.

The *P*-median model seeks, for a given number of facilities, to identify locations that minimize the total travelling distance from all clients to their closest serving facilities. As noted by Church and ReVelle [20], one important way to measure the effectiveness of a facility location is by determining the average distance traveled by those who visit it. With increasing average travelling distance, facility accessibility decreases, and thus the location's effectiveness decreases. This relationship holds for facilities such as libraries and schools, to which proximity is desirable. However, this model does not consider the "worst case" situation and so it may result in inequities, forcing a few remote clients to travel far.

The covering model finds the location of a given number of facilities that maximizes the total clients covered by these facilities within a maximum acceptable distance. The covering model is useful to allocate some facilities when minimizing the average distance traveled may not be appropriate. For example, emergency service facilities such as fire stations or ambulances need to be located within 15 minutes travelling time of every client. The critical nature of demands for service will dictate a maximum "acceptable" travelling distance or time. The covering problem model is widely used to determining the deployment of Emergency Medical Service System (EMS) vehicles in various settings [21,22].

The center model, for a given number of facilities, identifies a location arrangement that minimizes the maximum distance while requiring coverage of all clients. Unlike the covering model, which takes an input coverage distance, this model determines endogenously the minimal coverage distance associated with locating a given number of facilities. This model is useful when there are not enough facilities in reality while the service has to cover all the clients within a target region.

### Advanced facility location models for preventive health care facilities

Several methodologies for optimizing the configuration of preventive healthcare facilities have been recently proposed.

Verter and Lapierre [2] give a formalization of the preventive health care facility location problem. Their model is based on the covering model and considers the characteristics of preventive health care services by adding two constraints: (1) Probability of participation in a preventive program decreases with the distance between clients and their closet facility; (2) Each facility needs to have a minimal number of clients. They use a branch-and-bound based algorithm to identify optimal locations of facilities and to maximize participation in prevention programs. This is one of the main tools for finding the optimal solution of facility location problems [23].

Zhang et al.[3] extend Verter and Lapierre's model by using a queue method to capture the level of congestion of facilities in terms of waiting and service time. The queue method represents a facility as a capacity queue. When a client enters a facility, he/she would be put into a queue waiting for the service until all the others in the queue in front of him/her have been served. The authors calculate the total (travelling, waiting and service) time required for receiving the preventive service and use the total time as the accessibility of preventive health care facilities. They assume that each client would seek the services from that facility that has the minimum expected total time. The probability of participation in a preventive program decreases with the expected total time rather than the distance to be traveled. Additionally, they provide four heuristic methods to find optimal facility locations and compare the differences in terms of accuracy and computational requirements.

Although each study mentioned above contains a relevant element and achieves satisfactory results for some real applications, all of them assume that people would seek services from the closest preventive healthcare facility (defined either by traveling distance or total service time), which conflicts with the first characteristic of the preventive health care program, which assumes that people have choices about which preventive health care facility to attend.

### Solution approaches to facility location models

Two types of approaches are used to solve the facility location models: the *exact solution approach *[24] and *heuristic approach *[25]. Because the facility location problem is NP-hard [26], attempting a solution consumes a large amount of computational resources. The exact solution approach, such as branch and bound, can produce the best solution but cannot handle models with large amounts of constraints and variables since this consumes unacceptable amounts of computational resources. In order to solve a model with large amounts of constraints and variables, a heuristic approach is developed. This can produce acceptable solutions with fewer computational resources but will not guarantee finding the best solution.

The most well-known algorithm based on the heuristic approach is the Interchange algorithm [15]. The basic idea of the Interchange algorithm is to relocate a facility from its site in the current solution to an unused site. If the relocation produces a better value for a facility location model, then the change is accepted and a new solution is generated. Otherwise, the relocation is cancelled. The search process is repeated until no better solution can be found after relocating every facility.

A large number of research approaches for accelerating the Interchange algorithm has been proposed [27-30]. Densham and Rushton [27] propose to pre-store location information in the three data structures: demand string, candidate string and allocation table. The core idea is to examine only a subset of demand nodes to update the value of facility location models whenever a change of facility locations occurs. The demand string is built for each client location (called demand node in their work). This lists all candidate locations that can serve the demand node within an acceptable travelling distance. The candidate string is built for each candidate location. It lists all of the demand nodes that can be served by the candidate location within an acceptable travelling distance. The allocation table records the distances from each demand node to closest and second closest candidate sites that are occupied by facilities. When one facility moves from one candidate site to another, demand nodes affected by the move can be identified from the candidate strings of the two candidate sites. The change value of the facility location model can then be determined by examining these demand nodes in the allocation table. The allocation table needs to be updated when a change is accepted.

Since the above data structures accelerate the Interchange algorithm by recording the closest distance between demand nodes and facilities, the algorithm cannot be directly used to solve a preventive health care facility location model, which assumes that people might not take the service from the closest facility.

### Measurement of regional availability and facility's workload

Besides the travelling distance and total service time, other methods have been developed to measure accessibility of preventive health care services. According to Joseph and Phillips [31], regional availability is an approach primarily used to measure the accessibility of health care services by finding Health Professional Shortage Areas (HPSA). The approach generally assumes that given a specific range for the service being offered at a facility, every resident within that range is a potential client of the service. The regional availability of health care services is defined as the ratio of the number of people living in a region to the number of health care facilities in that region. People living in a higher ratio region can more conveniently access the service. Regional availability has been successfully used in measuring the accessibility of primary health care services [17] as well as preventive health care services [32].

Luo and Wang [33] compare different methods for measuring regional availability and recommend the usage of the two-step floating catchment area (2SFCA) method proposed by Radke and Mu [34]. The travelling distance catchment area of a facility or a client is an area within travelling distance of the facility or client. The 2SFCA method is implemented in two steps. First, it computes a travelling distance catchment area of each facility and calculates a facility-to-client ratio *R*_*j *_of each facility by counting the number of the clients covered by the facility's catchment area. Second, it computes a travelling distance catchment area of each client and calculates the regional availability of each client by summing up all *R*_*j *_values of the facilities within the client's catchment area. However, the 2SFCA approach cannot be directly used for location decision since it does not explicitly deal with the distance effect. The 2SFCA considers that facilities have the same attraction to clients within their catchment areas regardless of their actual travelling distance. Thus, changing the location of facilities would only result in a change in the facility-to-client ratio *R*_*j *_of each facility. The total ratio between facilities and clients would not change as long as the number of facilities and clients are fixed. In this paper we extend the 2SFCA method by adding the distance factor for measuring the accessibility of preventive health care services.

For clients in the catchment areas of multiple facilities, the probability that a client visits each facility can be estimated by using a Huff-based competitive model [13]. The expression of the model is:(1)

Where *P*_*ij *_is the probability of a client at site *i *travelling to a facility *j*;

*S*_*j *_is the size of a facility *j*;

*T*_*ij *_is the travelling time/distance between site *i *and facility j;

*λ *is a parameter to reflect the effect of travelling time/distance.

By using the model, the number of the clients in each site going to a facility can be estimated by multiplying the number of clients on the site with the probability that the clients at the site travel to the facility. The workload of the facility is estimated by summing up the number of clients traveling to the facility from all sites.

## Methods

### Formulation of the problem

Given a set of population centers and a set of candidate sites for facilities, the *Preventive Health Care Facility Location *(PHCFL) problem is to identify optimal locations for the predefined number of preventive health care facilities that maximize participation. Since the major determinant of participation in a preventive program is the accessibility of health care services [3], this paper solves the PHCFL problem by optimizing the accessibility of preventive health care services to population centers. In the following, we first introduce how to calculate the accessibility of preventive health care services to each population center. Then, a bi-objective model is given for the location optimization.

For the purposes of clarity, the following definitions pertain:

*I *Set of population centers (*i *= 1, ..., |*I|*);

*P*_*i *_Number of clients in a population center *i*;

*J *Set of candidate sites for the location of preventive health care facilities (*j *= 1, ..., |*J*|);

*n *The predefined number of preventive health care facilities;

*y*_*j *_If a facility opens at the candidate site *j*, then *y*_*j *_= 1; Otherwise, *y*_*j *_= 0;

*n*_*j *_The facility that is the closest to a candidate site *j*, *n*_*j *_∈ *J*;

*d*_*ij *_Travelling distance between a population center *i *and a candidate site *j*;

*d*_0 _The travelling distance threshold of a catchment area;

*d *The travelling distance threshold to define the remote place;

*A*_*i *_Accessibility of preventive health care services at a population center *i*;

*W*_*min *_Minimum required workload of a facility.

#### Accessibility of preventive health care services

We define the accessibility of preventive health care services as an index to represent the level of convenience for each population center receiving the service. This can be calculated using the following two steps:

*Step 1*. For each candidate site *j*, search all the population locations that are within a travelling distance threshold from the candidate site *j *(that is, the catchment area of *j*), and compute the facility-to-client ratio *R*_*j*_, within the catchment area:(2)

Where *P*_*i *_is the number of the clients in a population center *i*.

*Step 2*. For each population center *i*, search all the facilities whose locations that are within the travelling distance threshold from a population center *i *(that is, the catchment area of *i*), and the sum up the inverse distance-weighted facility-to-client ratio *R*_*j*_.(3)

Constraint (a) requires the number of facilities to be equal to a predefined number *n*. Constraint (b) ensures that the population covered by each facility is beyond the minimum workload or that a facility is open in a remote place. In constraint (b), first we use the Huff-based competitive model to estimate the probability of a client in a population center *i *traveling to a candidate site *j *as . Compared with equation (1), *S*_*j *_is set to one since we assume the size of each preventive health care facility is the same. *λ *is set to one. Second, from the Huff-based model, the number of clients in a population center *i *traveling to a candidate site *j *is estimated by multiplying the number of clients in the population center *i *with the probability that the clients in the population center *i *traveling to the candidate site *j*. Therefore, the workload of the facility in a candidate site *j *is estimated by summing up the number of clients from all the population centers within the candidate site *j*'s catchment area. In addition, we use a predefined travelling distance *d *as a threshold for choosing remote places. For remote areas, the constraint of the minimum workload is not required. We define a place as remote if the distance from it to other facilities is over *d *(Usually *d *>>*d*_0_).

In Step 1, the facility-to-client ratio *R*_*j *_describes the regional availability of each facility. A higher ratio indicates that fewer clients share a facility, and vice-versa. Step 2 first adds the distance factor by multiplying the inverse distance with the facility-to-client ratio *R*_*j*_. This takes into account the fact that all the clients within a facility's catchment area do not share this facility equally, rather that usage decreases with distance from the facility; second, the accessibility to a population center is calculated by summing up the inverse distance-weighted facility-to-population ratios of the facilities within the population center's catchment area. This step satisfies the assumption that people may go to any facility as long as it is within an acceptable travelling distance, which is defined as the travelling distance threshold *d*_0_. In other words, for a given population center, the more facilities are within the acceptable travelling distance and the closer these facilities are to this population center, the higher possibility the clients in the population center access a preventive health care service.

#### A bi-objective model

For the optimal design of preventive health care programs, two important objectives should be considered, efficiency and coverage [35]. The *efficiency *objective aims to maximize social welfare by achieving an optimal arrangement of health care facilities. *Coverage *aims to serve more people within a target area. In the above definition, the clients in a population center *i *can access services as long as the value *A*_*i *_is not zero and a larger value of *A*_*i *_indicates a better accessibility at a population center *i*. In this paper, we achieve the efficiency objective by maximizing the sum of population weighted accessibility values (equation (3)). We achieve the coverage objective by maximizing the number of people within the acceptable travelling distance of at least one facility (equation (5)). Therefore, the PHCFL problem can be formalized as a bi-objective model, shown as equation (6).(4)(5)(6)

Where *α *is defined as a co-efficient for balancing the two objectives. The value of *α *is determined by the importance of each objective according to the requirements of real-world applications. If *α *= 0, then the objective focuses only on service efficiency pertaining to overloaded facilities in high density population areas. With an increase in the *α *value, increased attention is paid to service 'coverage'. If *α *= + ∞, then the objective is only to eliminate uneven accessibility, thereby making the analysis the same as for the covering model.

### Solution approach to the bi-objective model

We use the Interchange algorithm to solve the bi-objective model. Since the data structures proposed by Densham and Rushton [27] do not record the accessibility values, they cannot be directly used to solve the bi-objective model. To accelerate the Interchange algorithm, we build two new data structures: *population group *and *candidate string*. The rationale for building these two data structures is the same as the idea in Densham and Rushton [27], which is to accelerate the Interchange algorithm by examining only a subset of population centers to update the value of the bi-objective model whenever a change of facility locations occurs.

**Figure 1:**
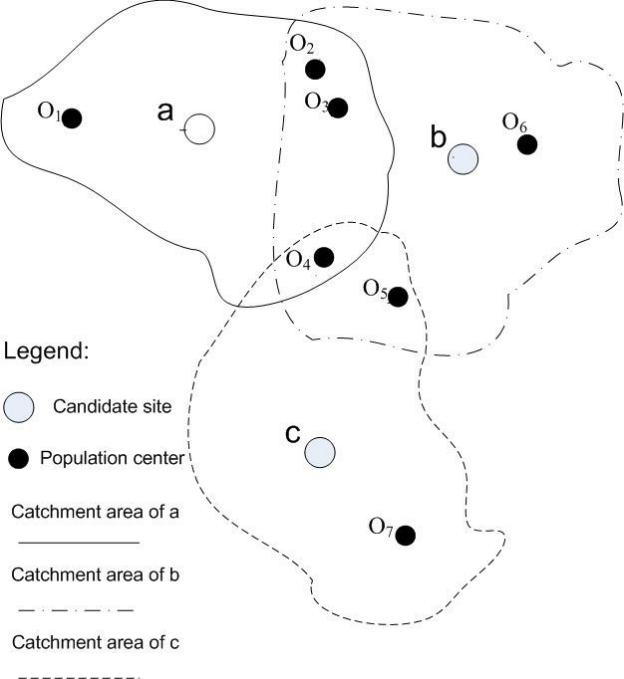
**Distribution of candidate sites and population centers**.

Population group is a data structure that aggregates similar population centers. Since the population centers in the same group are covered by the same set of candidate sites, they have the same accessibility value. For the example shown in Figure 1, Table 1 lists the population groups. Each population group records the candidate sites covering it and the potential population weighted accessibility value contributed from those candidate sites. For example, {*O*_*4*_} is covered by the catchment areas of *a*, *b *and *c*. According to equation (3), the accessibility value *A*_*4 *_of the population center *O*_*4 *_is . So, the potential population weighted accessibility value contributed from the candidate site *a *is ; from the candidate site *b *is ; from the candidate site *c *is , where *P*_*4 *_is the number of clients in the population center *O*_*4*_.

**Table 1:** Population group for the example in Figure 1.

Population group	Candidate site	Population weighted accessibility value
{O_1_}	a	

{O_2_, O_3_}	a	
	
	b	

{O_4_}	a	
	
	b	
	
	c	

{O_5_}	b	
	
	c	

{O_6_}	b	

{O_7_}	c	

A candidate string is built for every candidate site. The candidate string lists all of the population groups that can be covered by the candidate site. It is used to quickly find the population groups affected by the change of facility locations. As shown in Table 2, three candidate strings are built for the example in Figure 1. In the candidate string of the candidate site *a*, three population groups {*O*_*1*_}, {*O*_*2*_, *O*_*3*_} and {*O*_*4*_} are listed. Population centers {*O*_*2*_, *O*_*3*_}, {*O*_*4*_}, {*O*_*5*_} and {*O*_*6*_} are listed in the candidate string of the candidate site *b*. The candidate string of the candidate site *c *has three population centers: {*O*_*4*_}, {*O*_*5*_} and {*O*_*7*_}.

**Table 2:** Candidate string for the example in Figure 1.

Candidate string	Population group
a	{O_1_}, {O _2_, O_3_}, {O_4_}

b	{O_2_, O_3_}, {O_4_}, {O_5_}, {O_6_}

c	{O_4_}, {O_5_}, {O_7_}

When moving a facility from one candidate site to another, the change value of the bi-objective model (equation (6)) can be calculated by only examining the population groups listed under the candidate strings of the two sites. According to equation (6), the value of the bi-objective model includes the sum of population weighted accessibility values and the number of people covered by the facilities. The change of the total population weighted accessibility value that results from moving from one site to another can be calculated by subtracting the population weighted accessibility value contributed from one site by that of another. For example, a facility is changed from the candidate site *a *to *c*. The population groups listed in the candidate string of the candidate site *a *is {*O*_*1*_}, {*O*_*2*_, *O*_*3*_} and {*O*_*4*_}. From the population group data structure, we know that the population weighted accessibility value contributed from the candidate site *a *in population group {*O*_*1*_} is , population group {*O*_*2*_, *O*_*3*_} is , and population group {*O*_*4*_} is . Therefore, the population weighted accessibility value contributed by the candidate site *a *is . The population groups listed in the candidate string of the candidate site *c *is {*O*_*4*_}, {*O*_*5*_} and {*O*_*7*_}. The population weighted accessibility value contributed from the candidate site *c *in population group {*O*_*4*_}, {*O*_*5*_} and {*O*_*7*_} are ,  and , respectively. The population weighted accessibility value contributed from the candidate site *c *is . Thus, the change of the population weighted accessibility value from the candidate site *a *to *c *can be calculated by:

Similarly, the change in the number of people covered is the difference between the number of people covered by the original site and the number of people covered by the new site. For our example, the number of clients covered by *a *is *P*_1 _+ *P*_2 _+ *P*_3 _+ *P*_4_, and the number of clients covered by *c *is *P*_4 _+ *P*_5 _+ *P*_7_. So, when the facility location moves from *a *to *c*, the change of the number of clients covered is (*P*_4 _+ *P*_5 _+ *P*_7_) - (*P*_1 _+ *P*_2 _+ *P*_3 _+ *P*_4_).

Compared to the data structures in [27], the population group and candidate string do not need to be updated after facility locations change. The reason is that, given an acceptable traveling distance threshold, the catchment area of each candidate site and population center do not change. Neither the number of facilities in a population center's catchment area nor the number of population centers in a candidate site's catchment area change.

## Results and discussion

In this section, we apply our method to a real-world application, the breast cancer screening program in Alberta, Canada.

### Problem statement and data issues

Breast cancer is the most common cancer among Canadian women. In 2009, an estimated 22,700 Canadian women will be diagnosed with breast cancer and 5,400 would die from the disease; one in 9 women is expected to develop breast cancer during her lifetime and one in 28 will die from it [36]. Evidence from randomized controlled trials supports the recommendation that women aged 50 to 69 years be screened with annual or biennial mammography to reduce their risk of dying from breast cancer [37]. A population-based program to increase the number of Alberta women screened regularly for breast cancer was implemented in 1990 and today the Alberta Breast Cancer Screening Program (ABCSP) recommends Alberta women between the ages of 50 and 69 have a screening mammogram at least once every two years [38]. A key challenge is to determine the optimal number of screening facilities and their locations.

Our research considers the demand for services as measured by population in target groups (women between the ages of 50 and 69) in various locations. Estimates of the target population (Alberta women aged 50 to 69 years) were derived from census data at the Dissemination Area (DA) level [39] from the 2006 Canadian census (Statistics Canada). There are 327830 women within the target age in Alberta. In order to calculate the distance between the DAs and the facilities, we used the Postal Code Conversion File (PCCF) [40] to estimate the location of the DAs. A total of 5180 DAs were used in the research. Their values range from 0 to 920.

**Figure 2:**
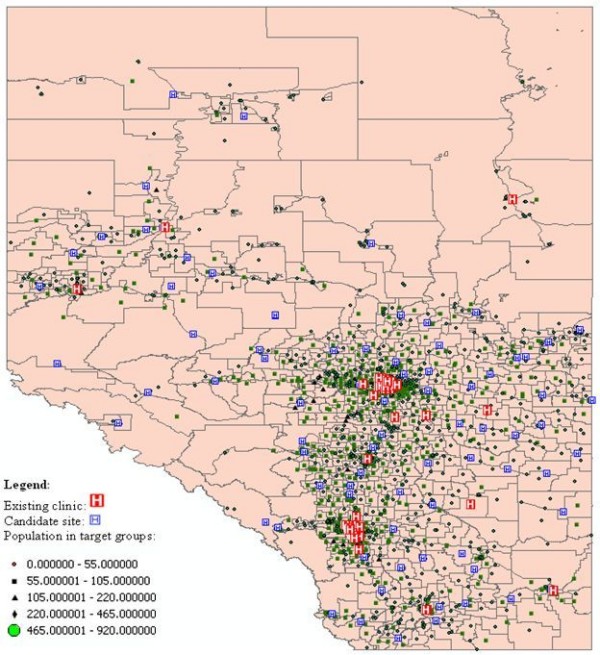
**Distribution of the supply and demand in Alberta breast cancer screening program**.

The existing 53 screening sites providing screening mammography in Alberta were extracted from the ABCSP. In addition, 92 candidate screening sites in Alberta were extracted from the Alberta Health Services website [41]. The candidate screening sites were defined as hospitals and cancer care facilities registered in Alberta but not used for breast cancer screening. The locations of clinics are geocoded to point locations using the GIS address matching technique [42]. Figure 2 shows the location of the DAs, the location of existing clinics, and the candidate sites for the screening service.

### Travelling distance and travelling time estimation

**Figure 3:**
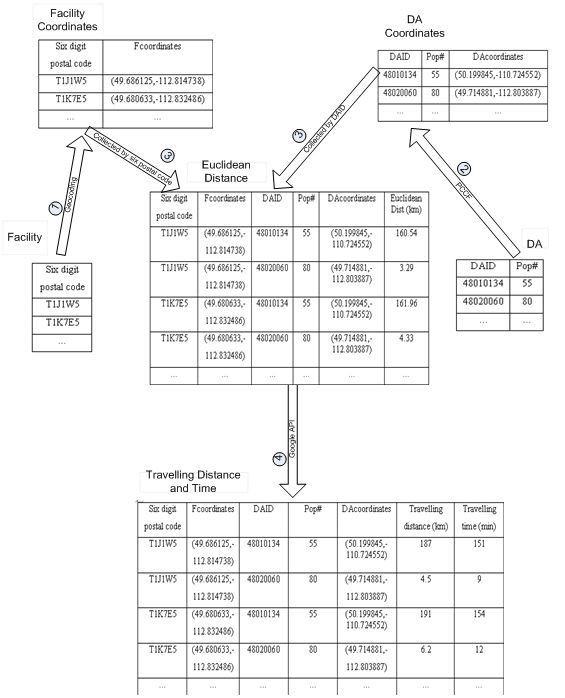
**Flow diagram of travelling distance and time estimation using the Google Maps API**.

**Figure 4:**
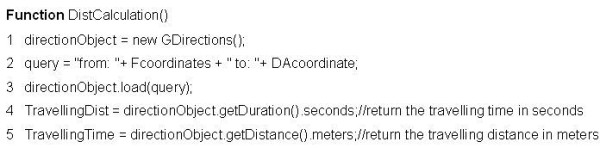
**Pseudo code for calculating the travelling distance and time by using Google API**.

In this subsection, we will briefly introduce how we use the Google maps API to estimate the travelling distance and travelling time between any pair of DA and facility. The process is comprised of four steps (as shown in Figure 3):

(1) Save the location information of facilities in the Facility Table as a Six digit postal code attribute. Create the Facility Coordinates Table by geocoding each six digit postal code in the Facility Table to the coordinates.

(2) Save the ID number and the population number of each DA in the DA Table. Create the DA Coordinates Table by using the PCCF to estimate the coordinates of each DA record in the DA Table.

(3) Create the Euclidean Distance Table by calculating Euclidean distance between any pair of the DA in the DA Coordinates Table and the facility in the Facility Coordinates Table.

(4) Create the Travelling Distance and Time Table by calculating the travelling distance and time between the DA and the facility in each record in the Euclidean Distance Table. The calculation is implemented in JavaScript[43] by calling the Google maps API. The pseudo code in Figure 4 shows how to calculate the travelling distance and time between one DA/Facility pair. First, an object instance called directionObject is created for the class GDirections in line 1. GDirections is a class defined in the Google Maps API and is used to obtain driving information and display these on a map. Second, the coordinates of the facility and the DA are uploaded as a string query using the function load()  in the GDirections class (lines 2-3). The load function extracts the coordinates from the string and sets the departure and destination location for the next step in the calculation. Finally, the travelling distance and time between the uploaded DA and facility are calculated by using the functions getDuration()  and getDistance() in the GDirections class (lines 4-5).

### Optimal facility configuration

In this subsection, our method is used to optimize the locations of screening clinics. Since the number of current screening sites in Alberta is 53, the predefined number of preventive health care facilities *n *is set to 53. The threshold travelling distance *d*_0 _of each facility is defined as thirty minutes driving time distance, a standard used by the U.S. Department of Health and Human Services for defining service areas [32]. Minimum required workload at each facility *W*_*min *_is set to 4000 according to the policy decision made by the Ministry of Health [2]. The predefined travelling distance for remote location *d *is set to 2*d_0_. The coefficient factor *α *in the objective model is equal to 30.

**Figure 5:**
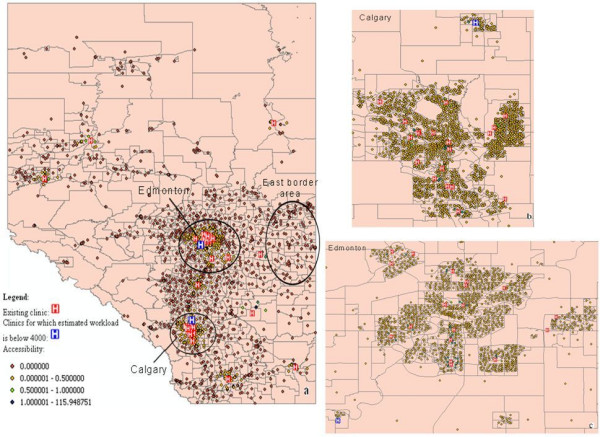
**Accessibility measurement on the existing facility configuration**. **a Alberta province. b Calgary metropolitan. c Edmonton metropolitan**.

Figure 5 shows the influence of the accessibility measurement on the existing facility configuration. The accessibility values of population centers range from 0 to 115.95. In Figure 5a, it is obvious that most screening clinics are located in two large metropolitan areas, Calgary and Edmonton while remote locations, such as the east border area, are lacking clinics. Figure 5b and 5c show the location of facilities in Calgary metropolitan and Edmonton metropolitan areas, respectively. Based on the workload estimation method mentioned above, one facility in north Calgary and one facility in southwest Edmonton cannot serve enough clients.

**Figure 6:**
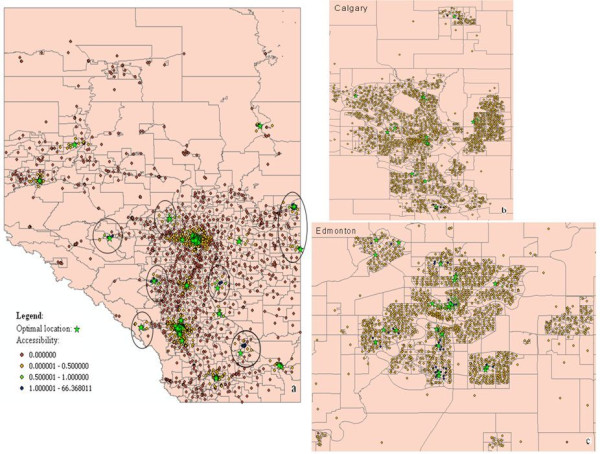
**Accessibility measurement on the optimal facility configuration**. **a Alberta province b Calgary metropolitan. c Edmonton metropolitan**.

Figure 6 shows the influence of the accessibility measurement on optimal facility configuration. The accessibility values of population centers range from 0 to 66.37. Compared with the existing facility configuration, the accessibility values in seven areas under the optimal facility configuration (shown in the circles in Figure 6a) dramatically higher. The facilities in Calgary metropolitan and Edmonton metropolitan areas are shown in Figure 6b and 6c respectively. In addition, all of the facilities have sufficient clients.

Table 3 compares the optimal facility configuration with the existing facility configuration based on average accessibility, coverage, and maximal accessibility. The *Average accessibility *records the average population weighted accessibility value of all population centers (i.e., ). The *Coverage *records the percentage of population that can access the service within the travelling distance threshold *d*_0 _(i.e., ). Table 3 shows that optimal facility configuration achieves better results in that it increases the average accessibility from 0.35 to 0.40 and improves the coverage from 78.42% to 81.86%. The value of maximal accessibility is smaller in the optimal facility configuration compared to that of the existing facility configuration because with our method some facilities in the high accessibility value area in the existing facility configuration are relocated to remote places. We also separate the accessibility value into different value segments and compare the number of people under the optimal facility configuration with the number of people under the existing facility configuration in each segment. People in the zero segment cannot be 'not covered' by any facility. The optimal facility configuration is better than the existing configuration because it reduces the number of people in this segment. People in the non-zero segment can be covered by at least one facility. People in higher value segments can get more convenient service. Compared with the existing facility configuration, the optimal facility configuration brings more people into higher value segments.

**Table 3:** Comparison between the existing facility configuration and the optimal facility configuration.

	Average accessibility	Coverage (%)	Maximal accessibility	Accessibility value segement			
				
				0	(0,0.5)	[0.5,1)	[1, max]
Existing configuration	0.35	78.42	115.95	70745	233700	7855	15530

Optimal configuration	0.40	80.35	66.37	64415	230385	14065	18965

### Parametric analyses

In this subsection, we perform sensitivity analyses on the impact of the following parameters in the real application.

**Figure 7:**
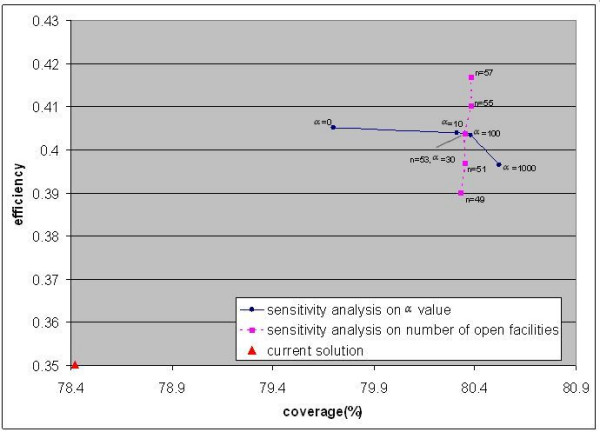
**Distribution of solutions**.

• *α *the coefficient factor in the objective function;

• *n *the predefined number of preventive health care facilities;

In Figure 7, we plot the optimal facility configurations on different parameters and the existing facility configuration into a solution space. Since we formalized the PHCFL problem as a bi-objective model, the solution space should have two dimensions: Y axis represents the efficiency (the average accessibility value of a facility configuration) and the X axis represents the coverage (the coverage value of that facility configuration). From Figure 7, two conclusions can be made. First, changing the value of *α *cannot improve the efficiency and coverage simultaneously. The optimal facility configurations denoted by dots show that with the increase of *α*, the efficiency of the optimal facility configuration decreases while the coverage of the optimal facility configuration increases. Second, with an increase in the predefined number of facilities allowed for a given facility configuration, both the efficiency and coverage of that configuration increase (denoted by squares). In addition, the optimal facility configuration can produce higher efficiency and coverage value with just 49 facilities, rather than with the existing configuration of 53 facilities.

## Conclusions and future research

This paper presents a method for locating preventive health care facilities so as to maximize participation. Assuming that the accessibility of a preventive health care service is a major determinant of participation to that service, this paper formalizes and solves the preventive health care facility location problem by optimizing the accessibility of preventive health care service. Unlike the traditional methods which measure the accessibility based only on distance, this paper defines a new accessibility measurement that combines the two-step floating catchment area method, the distance factor and the Huff-based model. The new accessibility measurement is suitable for preventive health care services because it considers two unique characteristics of preventive health care services. It also proposes a bi-objective model for performing location optimization. The bi-objective model is solved by the Interchange algorithm. To accelerate the solving process, we implement the Interchange algorithm by using population group and candidate string. In addition, this paper estimates the travelling distance and travelling time accurately by calling the Google Maps API. Experiments show that our work improves the performance of the Alberta breast cancer screening program.

Several extensions to our method are worth further investigation. First, in our method, the Interchange algorithm is implemented by following the idea proposed by Densham and Rushton [27]. While this can dramatically speed up the solving process, the accuracy is not improved. Recently, some meta-heuristic algorithms, such as VNS (Variable Neighborhood Search) [44] and Tabu [45], have been developed to improve optimization accuracy. Therefore, it would be interesting to incorporate strategies from meta-heuristic algorithms in order to increase accuracy. Second, there is a need for analyzing screening records of breast cancer in order to understand disease patterns. The disease patterns would help us to set the factors in the method precisely, such as the travelling distance threshold *d*_0_. Finally, Lapierre et al.[46] suggest that the use of satellite or mobile facilities might constitute an effective approach for improving participation of preventive health care programs. Indeed, the ABCSP has a program of mobile mammography sites that extends the reach of mammography services to Alberta women living in rural communities. Thus, extending the current location model to a hierarchical location model by considering both fixed and mobile facilities is meaningful.

## Competing interests

The authors declare that they have no competing interests.

## Authors' contributions

WG participated in the conceptualization of the study, designed the methodology, gathered the data and implemented the experiments.  XW participated in the conceptualization of the study, designed the methodology, gathered the data and supervised the experiments.  SEM participated in the conceptualization of the study and gathered the data.  All authors read and approved of the final manuscript.
